# Supplementary material for: Distinct beta frequencies reflect categorical decisions
Source: Nat Commun. 2023 May 22;14:2923. doi: 10.1038/s41467-023-38675-3 (PMC10203257; doi:10.1038/s41467-023-38675-3)
Supplement: Supplementary file 1 — Supplementary Information [file 41467_2023_38675_MOESM1_ESM.pdf]

**Monkey 1 dlPFC**	mean (+/- SD) prestimulus	mean (+/- SD) decision	decision > prestimulus (t)	decision > prestimulus (p)
frequency (Hz)	24.8 (+/- 1.8)	28.6 (+/- 2.3)	23.3	<1e-12
burst frequency (Hz)	22.3 (+/- 1.5)	25.1 (+/- 2.3)	14.4	<1e-12
burst number	1.44 (+/- 0.23)	1.76 (+/- 0.33)	18.9	<1e-12
burst peak power (a.u.)	3.71 (+/- 0.21)	4.04 (+/- 0.31)	15.9	<1e-12
burst time span (s)	158 (+/- 19)	194 (+/- 38)	12.1	<1e-12
burst frequency span (Hz)	6.35 (+/- 0.63)	7.12 (+/- 0.96)	10.1	<1e-12

Table S1. Beta frequency shift and burst profile during prestimulus vs decision delay in Monkey 1 dlPFC. t-values are results of two-sided t-tests, no adjustments for multiple comparisons, all t-value df = 198.

**Monkey 1 preSMA**	mean (+/- SD) prestimulus	mean (+/- SD) decision	decision > prestimulus (t)	decision > prestimulus (p)
frequency (Hz)	24.1 (+/- 1.5)	26.8 (+/- 3.2)	12.7	<1e-12
burst frequency (Hz)	22.1 (+/- 1.3)	23.7 (+/- 2.1)	14.5	<1e-12
burst number	1.62 (+/- 0.21)	2.04 (+/- 0.31)	30.3	<1e-12
burst peak power (a.u.)	3.71 (+/- 0.24)	4.13 (+/- 0.50)	13.1	<1e-12
burst time span (s)	170 (+/- 17)	193 (+/- 27)	10.8	<1e-12
burst frequency span (Hz)	6.81 (+/- 0.58)	7.31 (+/- 0.81)	9.0	<1e-12

Table S2. Beta frequency shift and burst profile of prestimulus vs. decision delay beta activity in Monkey 1 preSMA. t-values are results of two-sided t-tests, no adjustments for multiple comparisons, all t-value df = 198.

**Monkey 2 preSMA**	mean (+/- SD) prestimulus	mean (+/- SD) decision	decision > prestimulus (t)	decision > prestimulus (p)
frequency (Hz)	21.0 (+/- 0.8)	24.0 (+/- 1.9)	6.8	3e-6
burst frequency (Hz)	21.4 (+/- 1.0)	26.2 (+/- 2.2)	12.8	4e-10
burst number	1.37 (+/- 0.22)	1.06 (+/- 0.29)	-6.1	1e-5
burst peak power (a.u.)	3.81 (+/- 0.20)	4.43 (+/- 0.53)	4.7	2e-4
burst time span (ms)	194 (+/- 19)	127 (+/- 18)	-15.6	1e-11
burst frequency span (Hz)	6.63 (+/- 0.47)	4.51 (+/- 1.05)	-7.3	1e-6

Table S3. Beta frequency shift and burst profile of prestimulus vs. decision delay beta activity in Monkey 2 preSMA. t-values are results of two-sided t-tests, no adjustments for multiple comparisons, all t-value df = 17.

**dlPFC**	short trials (Hz) mean (+/-SD)	long trials (Hz) mean (+/-SD)	short > long (T(df))	short > long (p)
Monkey 1 - T1	30.0 (+/- 2.0)	29.2 (+/- 1.4)	2.6 (59)	.012
Monkey 1 - T2	29.9 (+/- 1.8)	28.0 (+/- 1.3)	9.0 (56)	2e-12
Monkey 1 - T3	29.9 (+/- 1.7)	28.0 (+/- 1.2)	8.9 (44)	2e-11
Monkey 1 - S2	29.3 (+/- 1.4)	28.2 (+/- 1.3)	5.2 (36)	8e-6

Table S4. Beta frequency shift in dlPFC during decision delay for trials categorized as “short” vs. “long”. T1, T2, and T3 are the interval categorization tasks with the shortest, middle, and longest sets of intervals (see Figure 1b for exact values), respectively. S2 is the distance categorization task with the middle set of distances (see Figure 1b for exact values). t-values are results of two-sided t-tests, no adjustments for multiple comparisons.

**preSMA**	short trials (Hz)<br>mean (+/-SD)	long trials (Hz)<br>mean (+/-SD)	short > long<br>(t(df))	short > long (p)
Monkey 1 - T1	30.1 (+/- 2.2)	28.1 (+/- 2.0)	5.4 (59)	1e-6
Monkey 1 - T2	29.3 (+/- 2.4)	27.7 (+/- 1.8)	4.2 (56)	1e-4
Monkey 1 - T3	29.0 (+/- 2.5)	27.4 (+/- 1.6)	3.3 (43)	.002
Monkey 1 - S2	28.7 (+/- 2.0)	27.3 (+/- 1.7)	4.7 (35)	4e-5
Monkey 2 - all tasks	25.2 (+/- 2.7)	23.6 (+/- 2.8)	2.7 (14)	.016

Table S5. Beta frequency shift in preSMA during decision delay for trials categorized as "short" vs. "long". T1, T2, and T3 are the interval categorization tasks with the shortest, middle, and longest sets of intervals, respectively (see Figure 1b for exact values). S2 is the distance categorization task with the middle set of distances. For Monkey 2, tasks additionally included S1 and S3, the distance categorization tasks with the shortest and longest distances, respectively. t-values are results of two-sided t-tests, no adjustments for multiple comparisons.

**Monkey 1 dlPFC**	mean (+/- SD)<br>short trials	mean (+/- SD)<br>long trials	short > long (t)	short > long (p)
burst frequency (Hz)	28.7 (+/- 1.1)	27.6 (+/- 1.0)	10.8	<1e-12
burst number	1.73 (+/-0.38)	1.8 (+/- 0.34)	-2.1	.039
burst peak power (a.u.)	3.95 (+/- 0.30)	4.07 (+/- 0.39)	-3.4	6e-4
burst time span (ms)	194 (+/- 51)	203 (+/- 45)	-1.8	.070
burst frequency span (Hz)	6.84 (+/- 1.26)	7.50 (+/- 1.10)	-5.5	8e-8

Table S6. Beta burst profile in dlPFC during decision delay for trials categorized as "short" vs. "long". t-values are results of two-sided t-tests, no adjustments for multiple comparisons, all t-value df = 198.

**Monkey 1 preSMA**	mean (+/- SD) short trials	mean (+/- SD) long trials	short > long (t)	short > long (p)
burst frequency (Hz)	28.4 (+/- 1.0)	27.4 (+/- 0.9)	9.9	<1e-12
burst number	2.02 (+/- 0.33)	2.05 (+/- 0.32)	-0.8	.400
burst peak power (a.u.)	4.04 (+/- 0.43)	4.19 (+/- 0.43)	-3.5	5e-4
burst time span (ms)	193 (+/- 37)	202 (+/- 33)	-2.4	0.018
burst frequency span (Hz)	7.24 (+/- 1.07)	7.69 (+/- 0.85)	-4.6	5e-6

Table S7. Beta burst profile in Monkey 1 preSMA during decision delay for trials categorized as “short” vs. “long”. t-values are results of two-sided t-tests, no adjustments for multiple comparisons, all t-value df = 198.

**Monkey 2 preSMA**	mean (+/- SD) short trials	mean (+/- SD) long trials	short > long (t)	short > long (p)
burst frequency (Hz)	25.9 (+/- 1.5)	25.2 (+/- 1.4)	3.1	.006
burst number	1.00 (+/- 0.29)	0.97 (+/- 0.41)	-0.5	.594
burst peak power (a.u.)	4.68 (+/- 0.71)	4.59 (+/- 0.74)	0.8	.436
burst time span (ms)	114 (+/- 15)	128 (+/- 31)	-2.0	.061
burst frequency span (Hz)	3.93 (+/- 1.23)	4.37 (+/- 1.22)	-2.6	.018

Table S8. Beta burst profile in Monkey 2 preSMA during decision delay for trials categorized as “short” vs. “long”. t-values are results of two-sided t-tests, no adjustments for multiple comparisons, all t-value df = 17.

**Monkey 1 dlPFC**	long trials (Hz) mean (+/- SD)	short trials (Hz) mean (+/- SD)	short > long (t (df))	short > long (p)
450ms	29.1 (+/- 1.9)	30.2 (+/- 2.1)	2.6 (99)	.011
500ms	29.1 (+/- 1.6)	30.1 (+/- 2.0)	2.9 (99)	.005
870ms	28.5 (+/- 1.6)	30.1 (+/- 1.7)	4.4 (81)	4e-5
920ms	28.9 (+/- 2.0)	30.5 (+/- 1.5)	6.42 (82)	8e-9

Table S9. Beta frequency shift in dlPFC during decision delay of the duration categorization tasks, for trials with identical stimuli within different task versions (i.e., trials with identical stimuli categorized as “short” in one task version but “long” in another; see overlapping stimuli outlined in Figure 1b). t-values are results of two-sided t-tests, no adjustments for multiple comparisons.

**Monkey 1 preSMA**	long trials (Hz) mean (+/- SD)	short trials (Hz) mean (+/- SD)	short > long (t (df))	short > long (p)
450ms	29.2 (+/- 2.5)	30.1 (+/- 2.1)	1.99 (99)	.049
500ms	28.9 (+/- 2.0)	30.0 (+/- 2.3)	2.8 (99)	.006
870ms	28.2 (+/- 1.6)	29.3 (+/-2.3)	2.8 (81)	.007
920ms	28.4 (+/- 1.7)	29.6 (+/- 2.0)	2.9 (82)	.005

Table S10. Beta frequency shift in preSMA during decision delay of the duration categorization tasks, for trials with identical stimuli within different task versions (i.e., trials with identical stimuli categorized as “short” in one task version but “long” in another; see overlapping stimuli outlined in Figure 1b). t-values are results of two-sided t-tests, no adjustments for multiple comparisons.

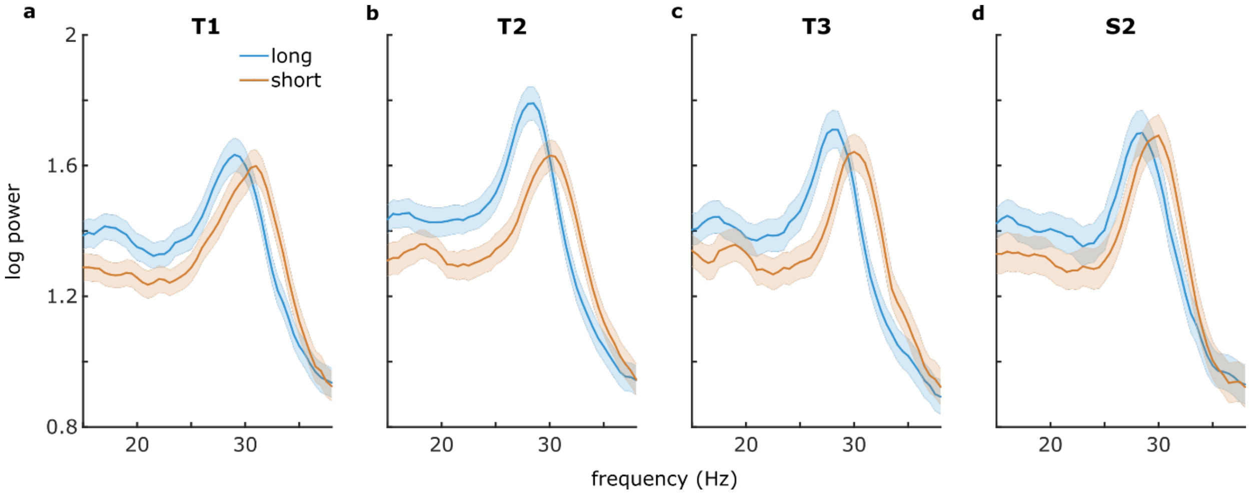

Figure S1. Beta peak frequency in monkey 1 dlPFC reflected the categorical decision during the decision delay in each version of the task. Power spectra for "long" stimulus (blue) vs. "short" stimulus trials (orange) during trials with correct responses **(a-c)** in the temporal categorization versions of the task with the shortest to longest stimuli respectively, and **(d)** the distance categorization task (see Figure 1b for exact values and Table S4 for statistics). Shaded regions around the line graphs represent the standard error of the mean.

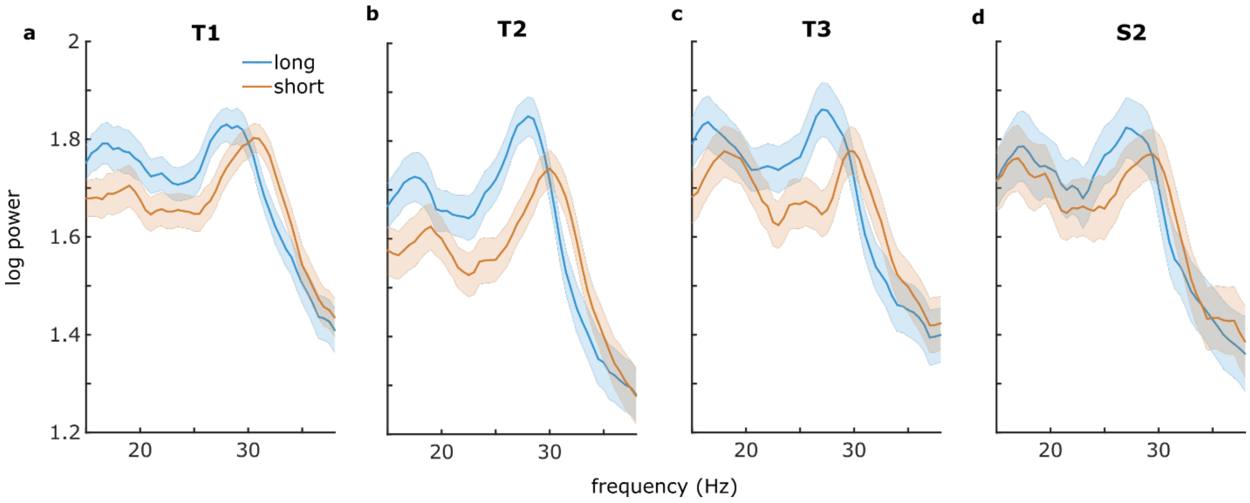

Figure S2. Beta peak frequency in monkey 1 preSMA reflected the categorical decision during the decision delay in each version of the task. Power spectra for "long" stimulus (blue) vs. "short" stimulus trials (orange) during trials with correct responses **(a-c)** in the temporal categorization versions of the task with the shortest to longest stimuli respectively, and **(d)** the distance categorization task (see Figure 1b for exact values and Table S5 for statistics). Shaded regions around the line graphs represent the standard error of the mean.

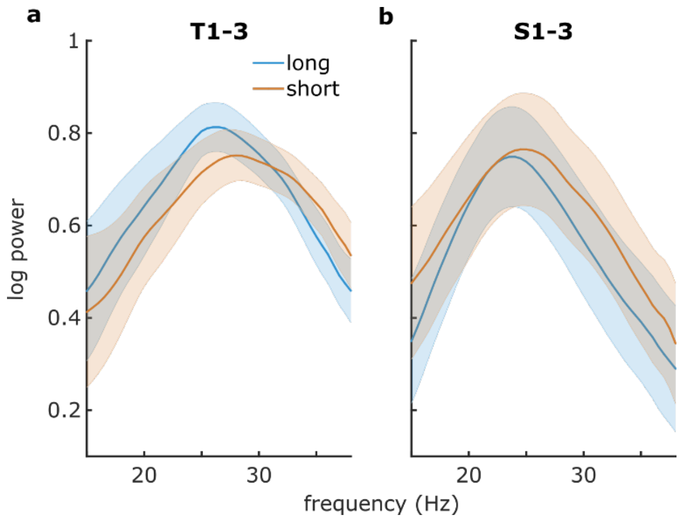

Figure S3. Beta peak frequency in monkey 2 preSMA reflected the categorical decision during the decision delay in each version of the task. Power spectra for "long" stimulus (blue) vs. "short" stimulus trials (orange) during trials with correct responses **(a)** in the temporal categorization versions of the task (pooled together) and **(b)** the distance categorization versions of the task (pooled together; see Figure 1b for exact stimulus values and Table S5 for statistics). Shaded regions around the line graphs represent the standard error of the mean.

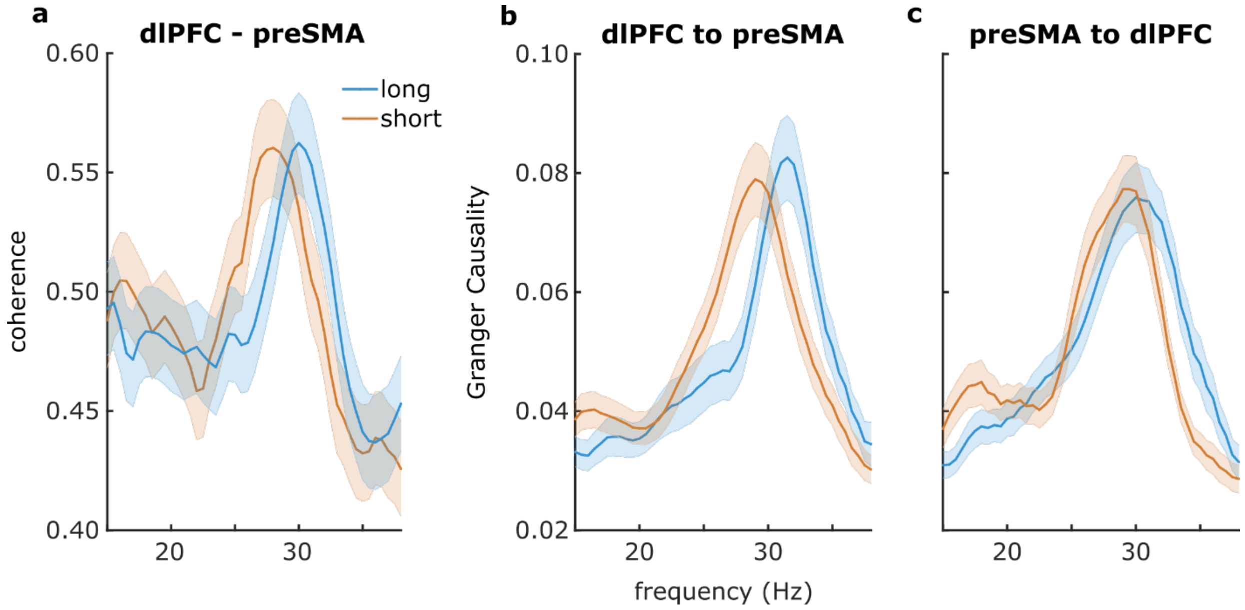

Figure S4. Peak frequencies of between-region connectivity reflected the categorical decision during the decision delay, even when the decision was incorrect. **(a)** Coherence between preSMA and dlPFC on incorrect trials. The direction of the peak frequency shift was

reversed compared with correct trials (see Figure 4a). **(b, c)** Granger causality between preSMA and dlPFC. The direction of the peak frequency of dlPFC to preSMA and that of preSMA to dlPFC Granger causality were reversed compared with correct trials (see Figure 4 c, d). However, on both correct and incorrect trials, peak frequency of dlPFC to preSMA, but not preSMA to dlPFC Granger causality, reflected the categorical decision. Shaded regions around the line graphs represent the standard error of the mean.

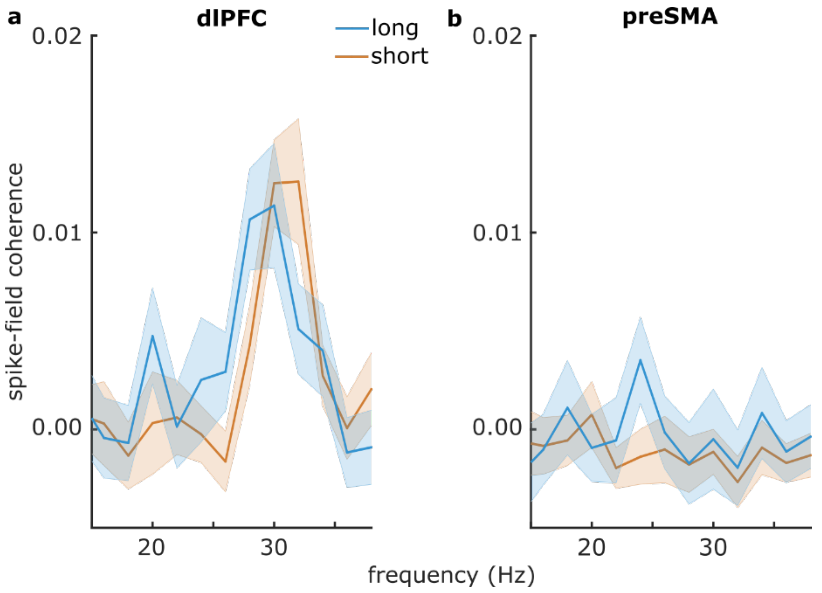

Figure S5. Peak frequency of spike-field coherence during the decision delay reflected the categorical decision in dlPFC but not preSMA. **(a)** Spike-field coherence in dlPFC between short-selective neurons and the LFP during short-categorized trials, and between long-selective neurons and the LFP during long-categorized trials: the peak frequency reflected the categorical decision. **(b)** Same for preSMA: there were no significant peaks in the beta range. Shaded regions around the line graphs represent the standard error of the mean.
