# Supplementary material for: Distinct beta frequencies reflect categorical decisions
Source: Nat Commun. 2023 May 22;14:2923. doi: 10.1038/s41467-023-38675-3 (PMC10203257; doi:10.1038/s41467-023-38675-3)
Supplement: Supplementary file 3 — Reporting Summary [file 41467_2023_38675_MOESM3_ESM.pdf]

Corresponding author(s): Hugo Merchant

Last updated by author(s): April 13 2023

Reporting Summary

Nature Portfolio wishes to improve the reproducibility of the work that we publish. This form provides structure for consistency and transparency in reporting. For further information on Nature Portfolio policies, see our Editorial Policies and the Editorial Policy Checklist.

Statistics

For all statistical analyses, confirm that the following items are present in the figure legend, table legend, main text, or Methods section.

n/a	Confirmed	
☐	☒	The exact sample size (n) for each experimental group/condition, given as a discrete number and unit of measurement
☐	☒	A statement on whether measurements were taken from distinct samples or whether the same sample was measured repeatedly
☐	☒	The statistical test(s) used AND whether they are one- or two-sided<br>*Only common tests should be described solely by name; describe more complex techniques in the Methods section.*
☒	☐	A description of all covariates tested
☐	☒	A description of any assumptions or corrections, such as tests of normality and adjustment for multiple comparisons
☐	☒	A full description of the statistical parameters including central tendency (e.g. means) or other basic estimates (e.g. regression coefficient) AND variation (e.g. standard deviation) or associated estimates of uncertainty (e.g. confidence intervals)
☐	☒	For null hypothesis testing, the test statistic (e.g. F, t, r) with confidence intervals, effect sizes, degrees of freedom and P value noted<br>*Give P values as exact values whenever suitable.*
☒	☐	For Bayesian analysis, information on the choice of priors and Markov chain Monte Carlo settings
☒	☐	For hierarchical and complex designs, identification of the appropriate level for tests and full reporting of outcomes
☒	☐	Estimates of effect sizes (e.g. Cohen's d, Pearson's r), indicating how they were calculated

Our web collection on statistics for biologists contains articles on many of the points above.

Software and code

Policy information about availability of computer code

Data collection	Visual Basic (Microsoft Visual Basic 6.0, 1998).
Data analysis	Fieldtrip toolbox (v. 20230118), Plexon Offline Sorter (v3.0, Plexon Inc. Dallas, TX, USA), Matlab 2019b (v. 9.7, The Mathworks Inc., Natick, Massachusetts, USA).<br>The code used to analyze the data supporting the claims of this study is publicly available here: https://osf.io/pza56/

For manuscripts utilizing custom algorithms or software that are central to the research but not yet described in published literature, software must be made available to editors and reviewers. We strongly encourage code deposition in a community repository (e.g. GitHub). See the Nature Portfolio guidelines for submitting code & software for further information.

Data

Policy information about availability of data

All manuscripts must include a data availability statement. This statement should provide the following information, where applicable:

- Accession codes, unique identifiers, or web links for publicly available datasets
- A description of any restrictions on data availability
- For clinical datasets or third party data, please ensure that the statement adheres to our policy

The data supporting the claims of this study are publicly available here: https://osf.io/pza56/

Human research participants

Policy information about studies involving human research participants and Sex and Gender in Research.

Reporting on sex and gender	N/A
Population characteristics	N/A
Recruitment	N/A
Ethics oversight	N/A

Note that full information on the approval of the study protocol must also be provided in the manuscript.

Field-specific reporting

Please select the one below that is the best fit for your research. If you are not sure, read the appropriate sections before making your selection.

☒ Life sciences ☐ Behavioural & social sciences ☐ Ecological, evolutionary & environmental sciences

For a reference copy of the document with all sections, see nature.com/documents/nr-reporting-summary-flat.pdf

Life sciences study design

All studies must disclose on these points even when the disclosure is negative.

Sample size	2 monkeys. This is the standard sample size in the field, and has been shown to be mathematically sufficient by Fries & Maris (2022), Journal of Cognitive Neuroscience. 34(7):114-118. This is also the sample size we had available, as this study is a reanalysis of existing data by Mendoza et al. (2018), Nature Communications 9, 1098.
Data exclusions	Due to hardware malfunctions while recording data from monkey 2, data collected after the malfunction were excluded.
Replication	The main finding was reached in two separate ways, with comparing peak frequencies and with analyzing instantaneous frequencies, both methods yielding the same results. Further, the main finding from monkey 1 was replicated in monkey 2.
Randomization	Within a recording session, the order of the experimental tasks was pseudo-randomized such that the order of tasks was counter-balanced at the end of the experiment.
Blinding	There was no group allocation, therefore blinding was not necessary.

Reporting for specific materials, systems and methods

We require information from authors about some types of materials, experimental systems and methods used in many studies. Here, indicate whether each material, system or method listed is relevant to your study. If you are not sure if a list item applies to your research, read the appropriate section before selecting a response.

Materials & experimental systems

n/a	Involved in the study	
☒	☐	Antibodies
☒	☐	Eukaryotic cell lines
☒	☐	Palaeontology and archaeology
☐	☒	Animals and other organisms
☒	☐	Clinical data
☒	☐	Dual use research of concern

Methods

n/a	Involved in the study	
☒	☐	ChIP-seq
☒	☐	Flow cytometry
☒	☐	MRI-based neuroimaging

Animals and other research organisms

Policy information about studies involving animals; ARRIVE guidelines recommended for reporting animal research, and Sex and Gender in Research

Laboratory animals	Two male Rhesus monkeys (Macaca mulatta), aged 6 and 8 years old at the time of data collection.
Wild animals	The study did not involve wild animals.
Reporting on sex	Both monkeys were male. This is reported in abstract and methods. The mechanisms involved are assumed to be common across sexes.
Field-collected samples	The study did not involve field-collected samples.
Ethics oversight	The experimental procedures were approved by the National University of Mexico Institutional Animal Care and Use Committee and conformed to the principles outlined in the Guide for Care and Use of Laboratory Animals (NIH, publication number 85–23, revised 1985).

Note that full information on the approval of the study protocol must also be provided in the manuscript.
